# Anti-Proliferative Activity of Meroditerpenoids Isolated from the Brown Alga *Stypopodium flabelliforme* against Several Cancer Cell Lines

**DOI:** 10.3390/md9050852

**Published:** 2011-05-13

**Authors:** David M. Pereira, Jose Cheel, Carlos Areche, Aurelio San-Martin, Juana Rovirosa, Luis R. Silva, Patricia Valentao, Paula B. Andrade

**Affiliations:** 1 REQUIMTE/Laboratory of Pharmacognosy, Department of Chemistry, Faculty of Pharmacy, Porto University, R. Anibal Cunha 164, 4050-047 Porto, Portugal; E-Mails: david.ffup@gmail.com (D.M.P.); luisfarmacognosia@gmail.com (L.R.S.); valentao@ff.up.pt (P.V.); 2 Department of Pharmacognosy, Faculty of Pharmacy, Charles University, Heyrovskeho 1203, 500 05 Hradec Kralove, Czech Republic; E-Mail: hornajosecarlos.cheel@faf.cuni.cz; 3 Department of Research and Development, CPN s.r.o., Dolni Dobrouc 401, 561 02 Dolni Dobrouc, Czech Republic; 4 Department of Chemistry, Faculty of Sciences, University of Chile, Casilla 653, Santiago, Chile; E-Mails: areche@uchile.cl (C.A.); aurelio@uchile.cl (A.S.-M.); jroviros@uchile.cl (J.R.)

**Keywords:** *Stypopodium flabelliforme*, meroditerpenoids, anti-proliferative, antimicrobial

## Abstract

The sea constitutes one of the most promising sources of novel compounds with potential application in human therapeutics. In particular, algae have proved to be an interesting source of new bioactive compounds. In this work, six meroditerpenoids (epitaondiol, epitaondiol diacetate, epitaondiol monoacetate, stypotriol triacetate, 14-ketostypodiol diacetate and stypodiol) isolated from the brown alga *Stypopodium flabelliforme* were tested for their cell proliferation inhibitory activity in five cell lines. Cell lines tested included human colon adenocarcinoma (Caco-2), human neuroblastoma (SH-SY5Y), rat basophilic leukemia (RBL-2H3), murine macrophages (RAW.267) and Chinese hamster fibroblasts (V79). Antimicrobial activity of the compounds was also evaluated against *Staphylococcus aureus*, *Salmonella typhimurium*, *Proteus mirabilis*, *Bacillus cereus*, *Enterococcus faecalis* and *Micrococcus luteus*. Overall, the compounds showed good activity against all cell lines, with SH-SY5Y and RAW.267 being the most susceptible. Antimicrobial capacity was observed for epitaondiol monoacetate, stypotriol triacetate and stypodiol, with the first being the most active. The results suggest that these molecules deserve further studies in order to evaluate their potential as therapeutic agents.

## 1. Introduction

Nature is an amazing source of chemical diversity and, as a consequence, naturally-derived compounds have unique pharmacological properties. This is easily observed if we consider that nearly 60% of all drugs introduced in therapy between 1981 and 2006 were first identified as natural products. This number rises to 75% if we only consider cancer drugs [1]. When compared with synthetic compounds, natural molecules usually have well-defined three-dimensional structures and fit biological targets that are conserved across species.

Products from marine origin have been increasingly used in human health, a trend that is likely to grow in the future [2]. Due to the harsh conditions that challenge marine organisms, such as low temperatures and light availability and high pressure, among others, marine organisms respond by synthesizing a number of secondary metabolites, some of them with potent pharmacological properties. In particular, algae have been referenced for producing molecules with amazing chemical diversity and, consequently, have interesting effects in humans. Molecules synthesized by algae show a number of different bioactivities, including anticancer [2–4], antibacterial [5] and immunomodulation [6], and are well documented for their use in an ethnopharmacological context.

Meroditerpenoids are a class of natural products consisting on a polyprenyl chain attached to a hydroquinone ring moiety and include plastoquinones, chromanols and chromenes. This class of compounds is common in brown algae and sometimes is used for taxonomic classification [7]. Brown algae (Phaeophyceae) produce a great variety of secondary metabolites, possessing many different skeletal types and biological activities.

**Figure 1.**
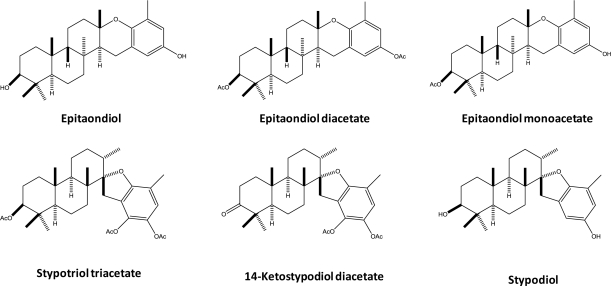
Structures of the meroditerpenoids evaluated in this study.

Due to the increasing prevalence and incidence of cancer in both developing and developed countries, new molecules to be used in cancer chemotherapy are needed. In this work, six meroditerpenoids (epitaondiol, epitaondiol diacetate, epitaondiol monoacetate, stypotriol triacetate, 14-ketostypodiol diacetate and stypodiol) (Figure 1) isolated from the brown alga *Stypopodium flabelliforme* were tested for their anti-proliferative properties against the cancer cell lines SH-SY5Y (human neuroblastoma), Caco-2 (human colorectal adenocarcinoma), RBL-2H3 (rat basophilic leukemia) and RAW.267 (mouse macrophages) and towards V79 non-cancer cell line (Chinese hamster fibroblasts). Few studies have addressed the potential biological activities of the meroditerpenoids studied herein. Soares *et al.* described the activity of epitaondiol against herpes simplex virus [8]. Epitaondiol diacetate was studied for its pharmacological effects in rat cardiovascular system and a negative inotropic effect of 35% was observed. A negative chronotropic effect was also noticed [9]. Epitaondiol and stypotriol triacetate revealed marked anti-inflammatory activities via decreased secretion of eicosanoids and modulation of the cyclooxigenase pathway through inhibition of some key enzymes, such as phospholipase A_2_, as assayed using [^3^H]oleate-labeled membranes of *Escherichia coli* [10].

The antimicrobial activity of these compounds has already been verified against several bacteria, namely *Bordetella bronchiseptica*, *Pseudomonas aeroginosa*, *Escherichia coli, Bacillus subtilis*, *Bacillus pumilus*, *Bacillus anthracis* and *Staphylococcus epidermidis*, and the results obtained showed moderate antimicrobial capacity [11]. Nevertheless, the study of their antimicrobial capacity was herein extended to other microorganisms.

## 2. Results and Discussion

### 2.1. Cell Proliferation Inhibition

Sulforhodamine B (SRB) is an aminoxanthene dye bearing two sulfonic groups, with a strong pink color. The SRB assay for cell proliferation inhibition testing relies on the ability of this molecule to bind to basic amino acids residues in acidic conditions and dissociate at basic pH. This binding is stoichiometric and thus, the amount of dye extracted from stained cells is directly proportional to the cell mass. For this reason, this test has been widely used for the evaluation of inhibition of cell proliferation [12].

In the work herein, meroditerpenoids from *S. flabelliforme* were tested for their ability to inhibit cell proliferation using a panel of human (Caco-2 and SH-SY5Y) and non-human (RBL-2H3 and RAW.267) cancer cell lines, as well as V79 non-cancer cells.

**Figure 2.**
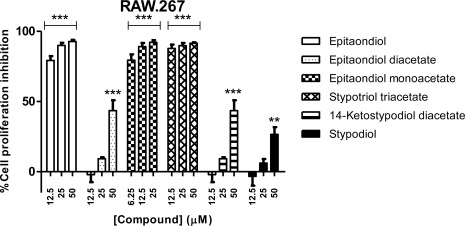
Effects of meroditerpenoids on the inhibition of cell proliferation in RAW.267 cells using the SRB assay. Values show mean + SE inhibition, as compared to negative control (0.1% DMSO), from three experiments performed in triplicate. ** *p* < 0.005; *** *p* < 0.0005.

**Figure 3.**
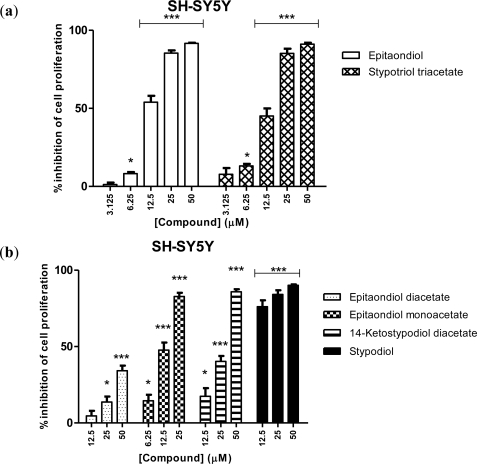
Effects of meroditerpenoids on the inhibition of cell proliferation in SH-SY5Y cells using the SRB assay. (**a**) Most and (**b**) less active compounds. For the most active (**a**), it was possible to test five different concentrations. Values show mean + SE inhibition, as compared to negative control (0.1% DMSO), from three experiments performed in triplicate. * *p* < 0.05; *** *p* < 0.0005.

**Figure 4.**
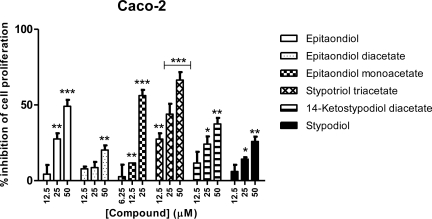
Effects of meroditerpenoids on the inhibition of cell proliferation in Caco-2 cells using the SRB assay. Values show mean + SE inhibition, as compared to negative control (0.1% DMSO), from three experiments performed in triplicate. * *p* < 0.05; ** *p* < 0.005; *** *p* < 0.0005.

**Figure 5.**
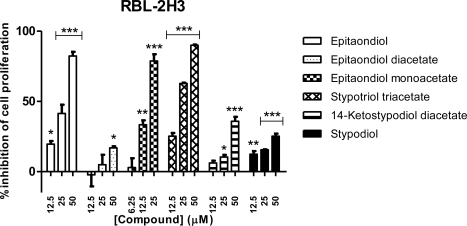
Effects of meroditerpenoids on the inhibition of cell proliferation in RBL-2H3 cells using the SRB assay. Values show mean + SE inhibition, as compared to negative control (0.1% DMSO), from three experiments performed in triplicate. * *p* < 0.05; ** *p* < 0.005; *** *p* < 0.0005.

**Figure 6.**
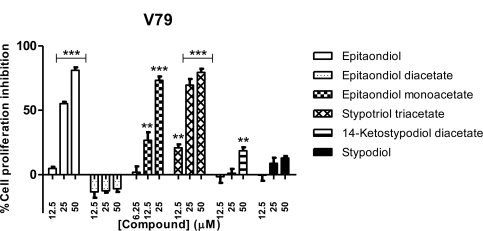
Effects of meroditerpenoids on the inhibition of cell proliferation in V79 cells using the SRB assay. Values show mean + SE inhibition, as compared to negative control (0.1% DMSO), from three experiments performed in triplicate. ** *p* < 0.005; *** *p* < 0.0005.

All cancer cells were affected by exposure to the compounds, although the several cell lines exhibited different sensitivities (Figures 2–6). Among the cell lines assayed, RAW.267 was one of the most affected and showed a significant inhibition of cell proliferation (Figure 2), with epitaondiol, epitaondiol monoacetate and stypotriol triacetate displaying nearly 100% inhibition at all tested concentrations. Epitaondiol diacetate, 14-ketostypodiol diacetate and stypodiol also displayed a concentration-dependant activity, although to a lesser extent (Figure 2). In fact, in general, epitaondiol, epitaondiol monoacetate and stypotriol triacetate were the most toxic compounds to all cell lines (Figures 2–6).

SH-SY5Y, a thrice-cloned neuroblastoma, originally from SK-N-SH, was the cell line displaying the highest susceptibility to the meroditerpenoids (Figure 3), on a par with RAW.267. Overall, with the exception of epitaondiol diacetate, all compounds exhibited nearly 100% inhibition of cell proliferation at the highest tested dose. IC_50_ of 12.2 μM and 14 μM were found for epitaondiol and stypotriol triacetate, respectively, which were the most active compounds (Figure 3a). The positive control used in this study, vincristine, yielded an IC_50_ of 0.03 μM. The neurotoxicity of meroditerpenoids has been observed before, but only 2β,3α-epitaondiol, an isomer of epitaondiol, was studied using a mouse cell line, neuro2a. With this mouse cell line, Sabry and colleagues [13] observed the effect of 2β,3α-epitaondiol and other meroditerpenoids and a LD_50_ between 2 and 11 μM was found [13]. To the best of our knowledge, this is the first time that the human neurotoxicity of these compounds is addressed. This cell line is particularly prone to damage caused by oxidative stress [14,15].

For Caco-2 cell line all compounds showed a concentration-dependent inhibitory effect, with stypotriol triacetate being the most active, followed by epitaondiol monoacetate and epitaondiol (Figure 4). A similar trend was found regarding RBL-2H3 cell line (Figure 5).

Regardless of the effect against cancer cells, cytotoxic compounds are interesting for human chemotherapy only when their toxicity is higher for cancer cells than for non-cancer ones. For this reason we tested the ability of the meroditerpenoids to inhibit cell proliferation of the non-cancer cell line V79 (Figure 6). Epitaondiol diacetate, 14-ketostypodiol diacetate and stypodiol revealed to have little or no effect on non-cancer V79 cell line, while against the cancer cell lines these same compounds displayed a selective activity (Figures 2–5). This selectivity against specific cancer cell lines is one of the requirements for the development of new drugs in order to lower the extent of side-effects. 14-Ketostypodiol diacetate has already proved to inhibit microtubules assembly and cell proliferation in DU-145 human prostatic cancer cells [16].

The results obtained in the present work point to the possible use of this class of compounds in a context of human chemotherapy against cancer. Nevertheless, further studies are required in order to understand the mechanism of action of these compounds.

### 2.2. Antimicrobial Activity

Compounds exhibiting antimicrobial properties for concentrations lower than 64 μg/mL are accepted as having notable antimicrobial activity [17], while those showing activity at concentrations below 10 μg/mL are considered as “clinically significant” [17,18].

Epitaondiol monoacetate, stypotriol triacetate and stypodiol showed some antimicrobial capacity, with the first displaying the major effect against gram-positive and gram-negative bacteria (MIC ≥ 114 μg/mL) (Table 1). *E. faecalis* appeared to be the most sensitive species. As for the remaining compounds, all bacteria were found to be resistant under the tested concentrations (Table 1). Thus, the compounds studied can hardly be considered good antimicrobial candidates.

## 3. Experimental Section

### 3.1. Compounds Isolation and Characterization

The brown alga *Stypopodium flabelliforme* was obtained from the underwater environment near Easter Island, Pacific Ocean. Isolation and characterization procedures were previously reported by us [19–21]. Generally, fresh algae were repeatedly extracted with dichloromethane. The extract was concentrated under reduced pressure and applied to a Sephadex LH-20 column. Because of the instability of the compounds, polar fractions were immediately acetylated with pyridine/acetic anhydride in order to avoid oxidation. Purification was done by thin layer chromatography (TLC) and column chromatography (CC) using grade silica gel. Complete spectroscopic characterization, including ^1^H NMR, ^13^C NMR and UV data has been published before for stypotriol triacetate, epitaondiol diacetate, epitaondiol monoacetate and epitaondiol [21], as well as for 14-ketostypodiol diacetate [20]. Stypodiol was obtained by hydrolysis of stypodiol diacetate previously isolated by the team [19]. Briefly, stypodiol diacetate (30 mg) was refluxed in basic aluminum oxide (1 mg) in benzene (30 mL) for 12 h [21]. Benzene was removed under reduced pressure and the residue dissolved in water (10 mL), extracted with dichloromethane (3 × 20 mL), dried, and evaporated to yield a solid (20 mg).

Stypodiol. ^1^H NMR (400 MHz, CDCl_3_) δ 6.42 (1H, brs), 6.40 (1H, brs), 3.25 (1H, dd, *J* = 11.6, 4.8), 3.21 (1H, d, *J* = 16.4), 2.75 (1H, d, *J* = 16.4), 2.18 (3H, s), 0.94 (6H, s), 0.86 (3H, s), 0.77 (3H, s), 0.67 (1H, d, *J* = 6.3). In agreement with literature [22].

Peak purity of all compounds was checked by HPLC analysis, using an HPLC unit (Gilson) and a Spherisorb ODS2 column (4.6 × 250 mm, 5 μm particle size). The eluent used was acetonitrile, at a flow rate of 0.9 mL/min. Detection was achieved with a Gilson diode array detector. Spectral data from all peaks were accumulated in the range of 200–400 nm, and chromatograms were recorded at 280 nm for all compounds, excepting sytpodiol, which was determined at 305 nm. The data were processed on a Unipoint Software system (Gilson Medical Electronics, Villiers le Bel, France). All tested compounds were >99% pure, excepting 14-ketostypodiol diacetate (>95%) and stypodiol (>80%).

### 3.2. Reagents

Sulforhodamine B (SRB), dimethylsulfoxide (DMSO) and vincristine were from Sigma-Aldrich (Steinheim, Germany). Dulbecco’s Modified Eagle Medium (DMEM), fetal bovine serum (FBS), phosphate buffer saline (PBS), human transferrin, fungizone and penicillin were from GIBCO (Invitrogen, UK). Tris-base, acetic acid and trichloroacetic acid were from Merck (Germany). Mueller Hinton Broth (MHB) and Mueller Hinton Agar (MHA) media were purchased from Liofilchem (Teramo, Italy). The water was treated in a Milli-Q water purification system (Millipore, Bedford, MA).

### 3.3. Cell Culture

Caco-2, V79, SH-SY5Y and RAW.267 cells were a kind gift from Professor Fernando Remião from the Laboratory of Toxicology, Faculty of Pharmacy, Porto University, Portugal. RBL-2H3 cells were obtained from ATTC. All cells were maintained in DMEM with 10% FBS and 1% penicillin, excepting RBL-2H3, which were cultivated in 15% FBS. V79 and Caco-2 cells were additionally added non-essential amino acids and, in the case of Caco-2, human-transferrin and fungizone. All cells were grown in an incubator at 37 °C and 5% CO_2_.

Stock solutions of all compounds in DMSO were prepared and diluted in media for testing. Cells were allowed to attach for 24 h, after which they were exposed to the compounds.

### 3.4. Sulforhodamine B Assay

The method by Houghton *et al.* [23], with modifications [24], was followed. Cells were plated with a density of 2 × 10^4^ (V79, RBL-2H3, Caco-2 and RAW.267) and 1.5 × 10^4^ (SH-SY5Y) cells/well and allowed to attach for 24 h at 37 °C, with 5% CO_2_. On the following day the medium was removed and cells were gently washed with warm PBS. Compounds in media were added and the plates were incubated for 48 h. The anticancer compound vincristine was used as a positive control.

After the incubation period media was removed, 100 μL of cold 40% trichloroacetic acid was added and plates were maintained at 4 °C for 60 min. Plates were then washed 5 times with tap water and allowed to dry. Afterwards, 50 μL of 0.4% SRB in 1% acetic acid were added and plates were incubated for 30 min.

After the incubation period plates were quickly washed with 1% acetic acid, in order to remove unbound dye, and allowed to dry. 100 μL of tris-base were added and after 10 min the plates were shaken.

Absorbance at 492 nm was determined in a multi-plate reader. OD values were plotted against concentration. Three independent assays were conducted, each one of them in triplicate. Values of inhibition of cell proliferation were expressed against a control of 0.1% DMSO.

### 3.5. Statistical Analysis

Comparisons of data from different groups *versus* control were performed by one-way ANOVA test, using GraphPad Prism 5. A *p* value lower than 0.05 was considered statistically significant.

### 3.6. Antimicrobial Activity

#### 3.6.1. Microorganisms

Six bacterial species were used for the experiments: *Staphylococcus aureus* (ATCC 20231), *Salmonella typhimurium* (ATCC 43971), *Proteus mirabilis* (ATCC 4479), *Bacillus cereus* (ATCC 31), *Enterococcus faecalis* (ATCC 20477) and *Micrococcus luteus* (ATCC 20030). Cultures were obtained from the Laboratory of Microbiology, Faculty of Pharmacy, Porto University, Portugal. Stock cultures were maintained on MHA at 4 °C. Bacterial inocula were prepared by growing cells in MHB for 24 h, at 37 °C. Cell suspensions were diluted in sterile MHB to provide initial cell counts of about 10^6^ CFU/mL.

#### 3.6.2. Antibacterial Effect

The minimum inhibitory concentrations (MIC) of the compounds studied herein were determined by two-fold serial dilution method, in 96-well plates. The compounds were dissolved in 5% (v/v) DMSO/MHB to a final concentration of 500 μM, excepting epitaondiol monoacetate, for which it was 250 μM due to solubility issues. Dilution series were prepared to obtain concentration ranges of 31.3–500 μM. 95 μL of MHB and 5 μL of suspension inoculum with 10^6^ CFU/mL were added in each well, which contained 100 μL of each compound’s solution. The negative control well consisted on 195 μL of MBH alone with 5% DMSO (v/v) and 5 μL of the standard inocula [25]. The plates were agitated using a plate shaker and incubated at 37 °C, for 24 h. The MIC of the tested compounds was detected following addition (40 μL) of 0.2 mg/mL *p*-iodonitrotetrazolium chloride and incubation at 37 °C for 30 min [26]. Viable microorganisms reduced the yellow dye to pink color.

MIC was defined as the lowest sample concentration that prevented dye’s change and exhibited complete inhibition of bacterial growth. The experiments were performed in duplicate and repeated independently three times.

## 4. Conclusions

In this work we evaluated the anti-proliferative ability of meroditerpenoids isolated from the brown alga *S. flabelliforme* against several cancer and non-cancer cell lines. All tested compounds inhibited the proliferation of cancer cells, but their activity against the non-cancer cell line was less marked.

The SH-SY5Y human neuroblastoma cell line showed higher susceptibility towards these meroditerpenoids than all of the other cell lines, thus pointing to the neurotoxicity of these secondary metabolites. Further studies are required in order to understand the mechanisms of action of these molecules and their potential application in cancer chemotherapy.

Low antimicrobial capacity was observed for epitaondiol monoacetate, stypotriol triacetate and stypodiol, with the first being the most active.

## Figures and Tables

**Table 1.** MIC of *S. flabelliforme* meroditerpenoids against selected bacteria.

**Bacteria**	**Alga compounds (μg/mL)**					
	**Epitaondiol**	**Epitaondiol diacetate**	**Epitaondiol monoacetate**	**Stypotriol triacetate**	**14-Ketostypodiol diacetate**	**Stypodiol**
**Gram-positive**						
*S. aureus*	>206	>249	>114	>277	>241	>206
*M. luteus*	>206	>249	>114	>277	>241	>206
*E. faecalis*	>206	>249	114	277	>241	206
*B. cereus*	>206	>249	>114	>277	>241	>206
**Gram-negative**						
*P. mirabillis*	>206	>249	>114	>277	>241	>206
*S. typhimurium*	>206	>249	>114	>277	>241	>206
